# N-myc and STAT interactor is a novel biomarker of severity in community-acquired pneumonia: a prospective study

**DOI:** 10.1186/s12931-022-02139-x

**Published:** 2022-09-19

**Authors:** Wanying Zhang, Hui Zhou, Mengyuan Cen, Wei Ouyang, Jie Chen, Lexin Xia, Xiuhui Lin, Jinliang Liu, Teng He, Feng Xu

**Affiliations:** Department of Infectious Diseases, The Second Affiliated Hospital, Zhejiang University School of Medicine, 88 Jiefang Road, Hangzhou, 310009 China

**Keywords:** N-myc and STAT interactor, Community-acquired pneumonia, Biomarker, Mortality

## Abstract

**Objectives:**

To tested the ability of N-myc and STAT interactor (NMI) levels in patients with community-acquired pneumonia (CAP) to predict the severity of the disease.

**Methods:**

Prospective observational analysis of patients with CAP was performed. The NMI levels in serum of 394 CAP patients on admission were measured by immunoassay. Thirty-day mortality and intensive care unit (ICU) admission were set as clinical outcomes. The predicting value of NMI for clinical outcomes was determined by receiver operating characteristic curve and logistic regression analysis. The internal validity was assessed using cross-validation with bootstrap resampling.

**Results:**

NMI was an independent risk factor for both 30-day mortality and admission to ICU for CAP patients. The area under curve (AUC) of NMI to predict mortality was 0.91 (95% CI: 0.86–0.96), and that to predict ICU admission was 0.92 (95% CI: 0.88–0.97), significantly higher than that of other biomarkers including procalcitonin and C-reactive protein. The proportion of clinical outcomes notably rose as NMI levels elevated (*P* < 0.001). The AUCs of the new score systems including NMI (N-PSI and N-CURB65 score) to predict outcomes were significantly higher than the original score systems.

**Conclusions:**

NMI ﻿is a novel biomarker for predicting CAP severity superior to former biomarkers in 30-day mortality and ICU admission.

**Supplementary Information:**

The online version contains supplementary material available at 10.1186/s12931-022-02139-x.

## Introduction

Community-acquired pneumonia (CAP) is a respiratory infection which can seriously threaten and affect human health, and can be accompanied by acute and severe inflammation within the lung [1, 2]. Prior studies have suggested that mild pneumonia has a good prognosis, but 21% of hospitalized CAP patients need intensive care unit (ICU) admission[3]. The mortality of severe CAP is estimated at 20–50% [4–6]. Previous studies have shown that delay in diagnosis of severe CAP leads to inappropriate therapeutic management of patients [7], thus increasing the length of stay (LOS) and mortality [8]. Therefore, early identification of patients with severe CAP is a vital issue.

The most commonly used tools for assessing the severity of CAP are the pneumonia severity index (PSI) and the British Thoracic Society (BTS) CURB65 score [9]. The PSI uses vital sign measurements (RR ≥ 30/min or temperature ≥ 40 °C), laboratory findings (pH < 7.35, blood urea nitrogen concentration ≥ 11 mmol/L, and sodium concentration < 130 mmol/l), patient history and age to predict 30-day mortality [10]. The CURB65 score is the recommended severity assessment strategy in the 2009 updated version of BTS guidelines [11]. CURB65 score system includes five indicators: altered mentation, blood urea > 7.0 mmol/l, respiratory rate ≥ 30/min, systolic blood pressure < 90 mmHg or diastolic blood pressure ≤ 60 mmHg, and age ≥ 65 years old. If two or more of these criteria are met, the pneumonia is classified as moderate to severe [12]. Biomarkers are also widely used for assessing the severity of CAP, including procalcitonin (PCT), C-reactive protein (CRP), and proadrenomedullin (proADM) [13–15]. In most studies of CAP severity evaluation tools, one of the most frequently used outcome is 30-day mortality [9]. However, some young severe CAP patients with good health in the past may have a low mortality but a high risk of ICU admission [16], so both the 30-day mortality and ICU admission should be considered when evaluating the efficacy of CAP severity tools.

N-myc and STAT interactor (NMI) is a transcriptional regulator of numerous nuclear signaling pathways [17]. It has been linked to macrophage activation as well as cancer growth and progression [18]. Several studies have shown a link between NMI and inflammation. NMI plays an important role in pro-inflammatory cytokine interleukin (IL)-32ε-mediated apoptosis regulating the host defense against pathogens such as *Mycobacterium tuberculosis* [19]. Degradation of NMI was enhanced by severe acute respiratory syndrome coronavirus (SARS-CoV) protein 6 by inhibiting the interferon (IFN) signal transduction pathway, ultimately promoting SARS-CoV survival in host cells [20]. Wang et al. found that after Sendai virus infection, NMI overexpression mice exerted antiviral effects by limiting the overproduction of type I IFN[21]. Similarly, Hu et al. found that prototype foamy virus replication was reduced by overexpression of NMI [22]. Ouyang et al. found that NMI exacerbates influenza A virus infection by increasing degradation of IRF7 through tripartite motif 21 [23]. Another study also showed that NMI activated macrophages and released proinflammatory cytokines [24]. Finally, serum NMI levels of were increased in patients died from severe infection [24]. These results indicate that NMI is involved in the pathogenesis of the inflammatory response, but do not define a relationship between NMI levels and CAP severity, and do not address the possibility of using NMI as a biomarker for disease severity.

In this study, we collected the samples of blood and bronchoalveolar lavage fluid (BALF) from CAP patients and measured NMI expression to explore whether the NMI can be used as a novel biomarker to stratify CAP severity and predict the prognosis of CAP.

## Materials and methods

### Participants

The study was carried out at the Second Affiliated Hospital of Zhejiang University (Hangzhou, Zhejiang, China) and was approved by the ethics committee. The serum was collected from 394 adult patients with CAP and 40 healthy controls, and BALF from 37 adult CAP patients and 23 controls. All samples were collected from the serum and BALF in the clinical laboratory of the Second Affiliated Hospital of Zhejiang University from January 2019 to November 2020. The flow diagrams for serum and BALF were shown in Additional file 1:  Fig. S1 and S2. Controls for BALF were defined as patients undergoing bronchoscopy due to lung tumors, pulmonary  nodules and other non-infectious diseases. The CAP and severe-CAP cohort were defined according to the 2007 Infectious Diseases Society of America (IDSA)/American Thoracic Society (ATS) guidelines [25]. The diagnostic process was performed blinded by two chief physicians. The exclusion criteria for this study were as follows: (1) patients under 18 years old; (2) patients with rheumatoid arthritis, systemic lupus erythematosus, and other autoimmune diseases etc.; (3) patients who have received chemotherapy, immunosuppressive agents, and hormone therapy within 4 weeks before the study; (4) patient who is using immunosuppressive medications or systemic hormone therapy in order to achieve the desired effect of immunosuppression (a dose of more than 10 mg/day of prednisone or other curative hormones), and continues to use these medications within 2 weeks before enrollment; (5) patients who have undergone a bone marrow transplant or peripheral blood stem cell transplant; (6) patients with HIV infection.

**Fig. 1:**
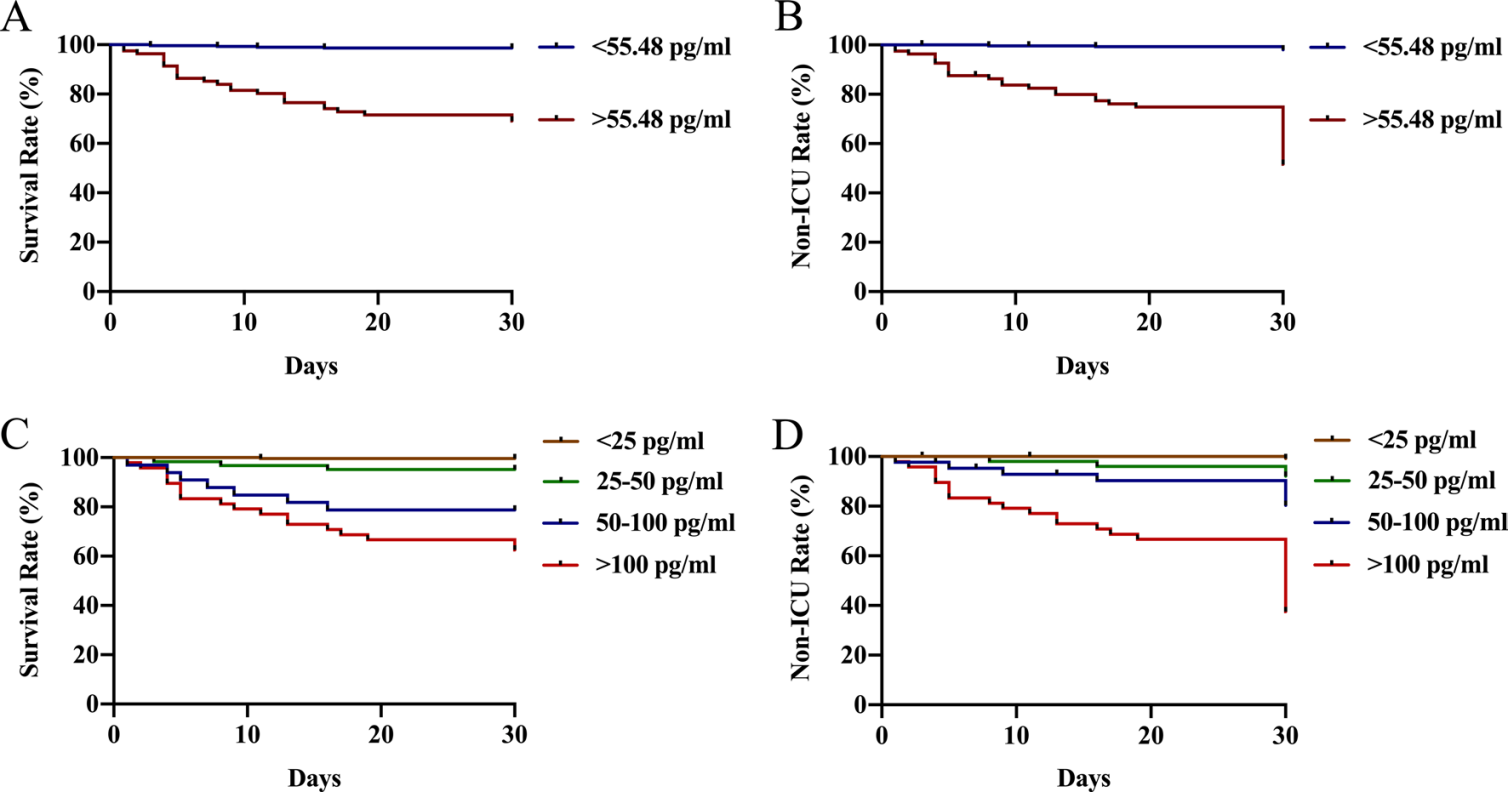
Kaplan–Meier survival curves by NMI cut-offs value and concentration stratification in CAP patients. Kaplan–Meier survival curves by NMI cut-offs value (55.48 pg/ml) for 30-day mortality **A** and ICU admission (**B**), and NMI concentration stratification (< 25 pg/ml, 25–50 pg/ml, 50–100 pg/ml, > 100 pg/ml) for 30-day mortality **C** and ICU admission (**D**)

**Fig. 2:**
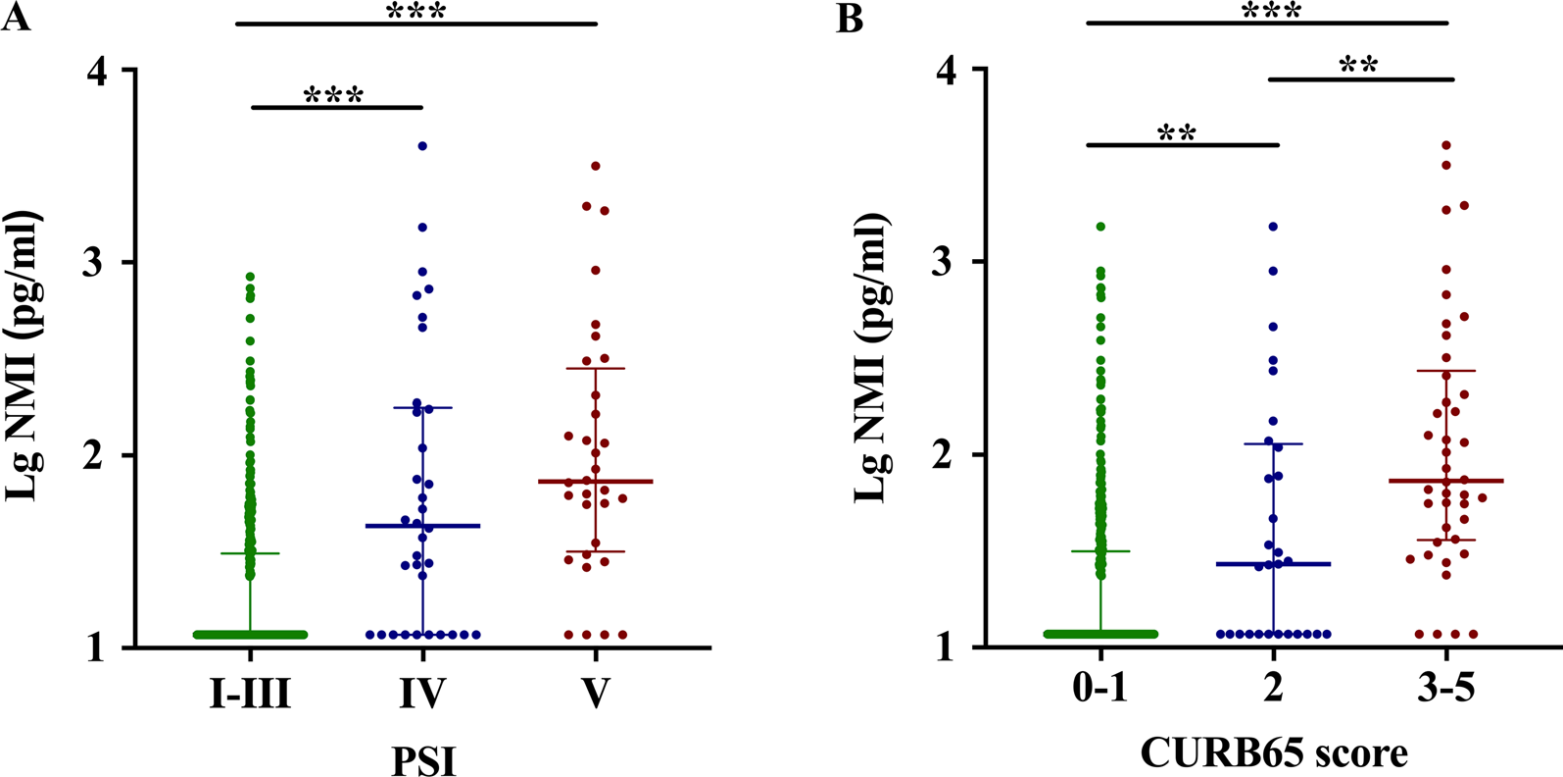
Distribution of NMI levels by PSI class **A** and CURB65 score (**B**). Lower and upper lines indicate the 25th and 75th percentiles; middle lines indicate the 50th percentiles. **: *P* < 0.01, ***: *P* < 0.001

### Clinical data collection

Clinical data were extracted from the electronic medical records of each patient. Coexisting illnesses (hypertensive heart disease, diabetes mellitus, congestive heart failure, renal dysfunction, chronic obstructive pulmonary disease, cerebrovascular disease, neoplastic disease, coronary artery disease and liver disease), the PSI and CURB65 score, PCT and CRP levels, white blood cell (WBC), neutrophil count, neutrophil count percentage (NCP) and radiographic characteristics on the day of admission, death and ICU admission within 30 days and LOS were recorded in CAP patients.

### Methods of measurement

Peripheral venous blood was collected within 24 h after admission to the hospital and BALF was gathered at the first bronchoscopy examination. Blood samples were kept at room temperature for four hours and centrifuged at 1000 rpm for 20 min at 4 °C. Aliquot of serum and BALF were kept at − 80 °C. The concentrations of NMI in serum were measured using ELISA kits (CSB-EL015893HU, CUSABIO) in accordance with the manufacturer’s instructions. Tests were perfomed in a blinded fashion. All samples were measured using serial dilutions to ensure measured concentrations were within the sensitivity range of the ELISA kit. The concentration of NMI in the stock solution below the lower limit were uniformly quantified as half the lower limit (11.72 pg/ml). A standard curve was performed for each ELISA plate to account for plate-to-plate variation in diagnostic sensitivity.

### Statistical analysis

We set 30-day mortality and ICU admission as clinical outcomes. A univariate analysis logistic regression analysis was carried out to screen potential predictors of outcomes with *P* values < 0.15. Independent risk factors for CAP severity were analyzed by a multivariate logistic regression model using variables from the univariate analyses. Subsequently, we assessed the differences of serum NMI and other parameters (PCT, CRP, WBC, neutrophil count and NCP) between groups that died or were admitted to the ICU and groups without these outcomes. We then used receiver operating characteristic (ROC) curve to evaluate the area under the curve (AUC) of NMI and other indexes for predicting occurrence of 30-day mortality and ICU admission, respectively. The cut-off value for biomarkers was set at the maximum value of Youden's index (sensitivity + specificity − 1) [26], the sensitivity and specificity of these biomarkers were also reported. To determine the model's internal validation predictive power, the bias-corrected AUC with 95% CI was calculated by cross-validation on the base of 1000 design matrix bootstrap replicates[27]. We further analyzed differences in the incidence of clinical outcomes among various NMI cutoffs by Kaplan–Meier survival curves. After that, we assessed the correlation of NMI with LOS and other biomarkers using Spearman correlation coefficient. Besides, we evaluated the diversities of NMI concentrations in different levels of the PSI and CURB65 score and then compared effectiveness of new severity score system (N-PSI and N-CURB65 score system) with the original score system for predicting clinical outcomes by ROC curve. Finally, we compared the levels of NMI in BALF between severe and non-severe groups of CAP patients.

All results were analyzed by SPSS-16 software and GraphPad Prism 8.0 (GraphPad software). Measurement data and enumeration data were expressed by median quartile spacing and frequency (percentage), respectively. All data were represented by scatter plots. Horizontal lines showed the lower quartile, median, and upper quartile. Mann–Whitney U test and Kruskal–Wallis H test were used to compare differences between two or more groups, and Nemenyi test was used for pairwise comparisons after multiple groups. Log-rank test was used to verify differences among Kaplan–Meier curves. We used method of DeLong et al. and Z-statistics to determine the difference of AUC between two and multiple ROC curves. All tests were two tailed and *P* value < 0.05 was regarded as statistically significant.

## Results

### Clinical characteristics

The serum cohort consisted of 394 CAP patients with a median age of 54 yrs (31–67). The characteristics of these patients is shown in Table 1. In the cohort, 59.6% (235/394) were male. 27.2% (107/394) of them had an antibiotic pretreatment before admission. The top three coexisting illnesses were hypertension (17.3%, 68/394), diabetes mellitus (9.1%, 36/394) and chronic obstructive pulmonary disease (4.3%,17/394). 8.6% (34/394) of patients had a PSI score IV and 32 patients (8.1%, 32/394) had a PSI score V, while 10.7% (42/394) were classified as high-risk patients based on CURB65 score (≥ 3 points). 7.9% (31/394) of the patients died within 30 days of admission and 11.7% (46/394) were admitted to the ICU. The median age of healthy controls in the serum cohort were 37 yrs (28–47) and 40% (16/40) were male. BALF was collected from 37 CAP patients and 23 non-infected patients. The characteristics of these patients from whom BALF was collected are shown in Additional file 1: Tables S1 and S2. The clinical basic characteristics comparing survivors/non-survivors and patients admitted/not admitted to the ICU are shown in Additional file 1: Tables S3 and S4.

**Table 1:** Baseline characteristics and outcomes of CAP patients from whom serum was collected

Characteristics	Patients with CAP (n = 394)
**Demographic characteristics**	
Age (years)	54 (31–67)
Males	235 (59.6)
**Coexisting illnesses**	
Hypertensive heart disease	68 (17.3)
Diabetes mellitus	36 (9.1)
Chronic obstructive pulmonary diseasee	17 (4.3)
Liver disease	14 (3.6)
Coronary artery disease	11 (2.8)
Renal dysfunction	9 (2.3)
Congestive heart failure	8 (2.0)
Cerebrovascular disease	8 (2.0)
Neoplastic disease	8 (2.0)
**Antibiotic pretreatment**	107 (27.2)
**Laboratory findings**	
PCT (ng/ml)	0.28 (0.15–0.59)
CRP (mg/l)	44.55 (15.98–106.25)
WBC (10^9/l)	7.95 (6.10–11.30)
Neutrophils (10^9/l)	6.00 (4.10–8.90)
NCP (%)	75.70 (67.08–83.65)
**Radiographic findings**	
Pleural effusion	107 (27.2)
Multilobar infection	146 (37.1)
**PSI class**	
I–III	328 (83.3)
IV	34 (8.6)
V	32 (8.1)
**CURB65 score class**	
0–1	323 (82.0)
2	29 (7.4)
3–5	42 (10.6)
**Clinical outcomes**	
30-day mortality	31 (7.9)
ICU admission	46 (11.7)

Data are presented as median (interquartile range) or n (%)

**Table 2:** Logistic regression analysis of serum parameters and severity score for predicting 30-day mortality and ICU admission

Variables	Prediction of mortality				Prediction of ICU admission			
	Univariate		Multivariate		Univariate		Multivariate	
	Odds ratio (95% CI)	*P* value	Odds ratio (95% CI)	*P* value	Odds ratio (95% CI)	*P* value	Odds ratio (95% CI)	*P* value
Age	1.048 (1.026–1.072)	** < 0.001**	1.032 (1.000–1.069)	0.064	1.040 (1.023–1.059)	** < 0.001**	1.022 (0.995–1.052)	0.117
Gender	1.702 (0.786–3.993)	0.194	–	–	2.327 (1.179–4.943)	**0.020**	1.655 (0.585–5.064)	0.354
NMI (pg/ml)	1.005 (1.003–1.007)	** < 0.001**	1.005 (1.003–1.008)	** < 0.001 **	1.010 (1.007–1.014)	** < 0.001**	1.008 (1.004–1.011)	** < 0.001**
PCT (ng/ml)	1.037 (1.019–1.059)	** < 0.001**	0.969 (0.928–1.003)	0.105	1.121 (1.070–1.198)	** < 0.001 **	1.058 (1.013–1.116)	**0.019**
CRP (mg/l)	1.012 (1.008–1.017)	** < 0.001**	1.008 (1.001–1.016)	**0.026 **	1.011 (1.008–1.015)	** < 0.001**	1.000 (0.993–1.007)	0.964
WBC (10^9/l)	1.037 (1.006–1.068)	**0.018**	1.201 (0.667–1.716)	0.371	1.065 (1.032–1.105)	** < 0.001**	1.035 (0.758–1.444)	0.829
Neutrophils (10^9/l)	1.058 (1.02–1.097)	**0.002**	0.780 (0.492–1.526)	0.327	1.088 (1.049- 1.133)	** < 0.001**	1.013 (0.653–1.536)	0.951
NCP (%)	1.111 (1.066–1.165)	** < 0.001**	1.080 (0.982–1.197)	0.121	1.083 (1.049–1.121)	** < 0.001**	1.024 (0.948–1.116)	0.562
PSI	3.117 (2.290–4.434)	** < 0.001 **	0.889 (0.468–1.705)	0.719	2.809 (2.181–3.709)	** < 0.001**	1.190 (0.664–2.162)	0.560
CURB65 score	3.336 (2.474–4.682)	** < 0.001**	2.882 (1.529–5.647)	**0.001 **	3.000 (2.323–3.972)	** < 0.001**	2.051 (1.167–3.664)	**0.013**

Gender is categorical variables (male set as 1 and female set as 0). Other indicators are continuous variables. The PSI and CURB65 score are ordered categorical variable that calculated as continuous variables (values are set according to their severity grades). The bold value reflects the* P* value < 0.05, and there is a significant difference

**Table 3:** Diagnostic performance analysis of serum parameters and severity score for CAP patients

	Prediction of mortality						Prediction of ICU admission					
	Cut-off value	AUC (95% CI)	AUC (95% CI) §	Sensitivity (%)	Specificity (%)	Youden index	Cut-off value	AUC (95% CI)	AUC (95% CI) §	Sensitivity (%)	Specificity (%)	Youden index
NMI (pg/ml)	55.48	0.91 (0.86–0.96)	0.91 (0.85–0.95)	87.10	85.12	0.72	55.48	0.92 (0.88–0.97)	0.92 (0.88–0.96)	86.96	88.22	0.75
PCT (ng/ml)	0.89	0.79** (0.70–0.88)	0.80 (0.70–0.88)	64.52	86.61	0.51	0.80	0.80** (0.72–0.88)	0.80 (0.73–0.88)	65.32	87.76	0.53
CRP (mg/l)	125.1	0.78** (0.69–0.87)	0.78 (0.68–0.86)	61.29	75.12	0.36	123.5	0.76*** (0.69–0.84)	0.76 (0.69–0.83)	56.51	85.30	0.42
WBC (10^9/l)	13.15	0.59*** (0.47–0.71)	0.59 (0.47–0.70)	35.48	84.81	0.20	14.85	0.58*** (0.48–0.69)	0.58 (0.47–0.68)	32.61	90.20	0.23
Neutrophils (10^9/l)	5.55	0.65*** (0.54–0.75)	0.65 (0.54–0.74)	80.65	45.73	0.26	11.20	0.63*** (0.53–0.72)	0.63 (0.52–0.72)	36.96	87.05	0.24
NCP(%)	77.15	0.78** (0.70–0.86)	0.78 (0.70–0.85)	90.32	58.84	0.49	81.45	0.73*** (0.65–0.82)	0.73 (0.65–0.82)	69.57	72.33	0.42
PSI	3.5	0.85^&&^ (0.77–0.94)	0.85 (0.77–0.92)	88.43	77.42	0.66	3.5	0.84^&&&^ (0.77–0.90)	0.84 (0.77–0.90)	65.22	89.66	0.55
N-PSI	3.5	0.91 (0.86–0.96)	0.91 (0.86–0.95)	86.78	83.87	0.71	2.5	0.90 (0.86–0.94)	0.90 (0.86–0.93)	91.30	74.71	0.66
CURB65 score	1.5	0.86^##^ (0.77–0.95)	0.86 (0.76–0.93)	83.87	87.60	0.71	1.5	0.84^###^ (0.76–0.91)	0.84 (0.76–0.90)	73.91	89.37	0.63
N-CURB65 score	2.5	0.93 (0.89–0.97)	0.93 (0.89–0.96)	83.87	92.84	0.77	1.5	0.92 (0.89–0.96)	0.92 (0.89–0.95)	80.43	86.49	0.67

N-CURB65: combined NMI with CURB65 score, N-PSI: combined NMI with PSI; *: *P* < 0.05, **: *P* < 0.01, ***: *P* < 0.001 compared with NMI using the z statistic; ^&&^: *P* < 0.01, ^&&&^: *P* < 0.001 compared with N-PSI using the nonparametric method of DeLong et al.; ^##^: *P* < 0.01, ^###^: *P* < 0.001 compared with N-CURB65 score using the nonparametric method of DeLong et al., §: Internally validated AUC with 95% CI by bootstrap

### Logistic regression analysis of NMI and other parameters to predict outcomes

Univariate analysis and multivariate binary logistic regression analysis were carried out to investigate the independent risk indexes in predicting 30-day mortality and ICU admission in CAP patients. Univariate analysis revealed that age, gender, NMI, PCT, CRP, WBC, Neutrophils, NCP, the PSI and CURB65 score were potential risk factors in predicting ICU admission (*P* < 0.15), and the above parameters except gender were also candidates for predicting 30-day mortality (*P* < 0.15), (Table 2). Possible risk factors were further calculated in a multivariate logistic regression analysis. According to the results, NMI, CRP and CURB65 score were independent risk factors for 30-day mortality and NMI, PCT, and CURB65 score were for ICU admission (*P* < 0.05), (Table 2).

### Predictive values of NMI for clinical outcomes in patients with CAP

30-day mortality and ICU admission were set as clinical outcomes representing the severity of CAP patients. Levels of NMI, CRP, PCT, neutrophil count and NCP were significantly increased in patients that died or were admitted to the ICU, but no significant difference was found in WBC (Additional file 1: Figs. S3 and S4). The AUC of NMI to predict mortality was 0.91 (95% CI: 0.86–0.96), much higher than that of PCT [0.79 (95% CI: 0.70–0.88), P < 0.01], CRP [0.78 (95% CI: 0.69–0.87), P < 0.01], WBC [0.59 (95% CI: 0.47–0.71), P < 0.001], neutrophil count [0.65 (95% CI: 0.54–0.75), P < 0.001], and NCP [0.78 (95% CI: 0.70–0.86), P < 0.01] (Table 3, Additional file 1: Fig. S3). The AUC of NMI to predict ICU admission was 0.92 (95% CI: 0.88–0.97) (Table 3, Additional file 1: Fig. S4). Furthermore, after internal validation, the AUCs of NMI to predict mortality and ICU admission were 0.91 (95% CI: 0.85–0.95) and 0.92 (95% CI: 0.88–0.96), respectively (Table 3), which were very close to the original AUCs before validation. At the best cut-off value of NMI (55.48 pg/ml), the Youden index for predicting 30-day mortality and ICU admission by NMI was 0.72 and 0.75, respectively, both higher than other serum indicators (Table 3).

**Fig. 3:**
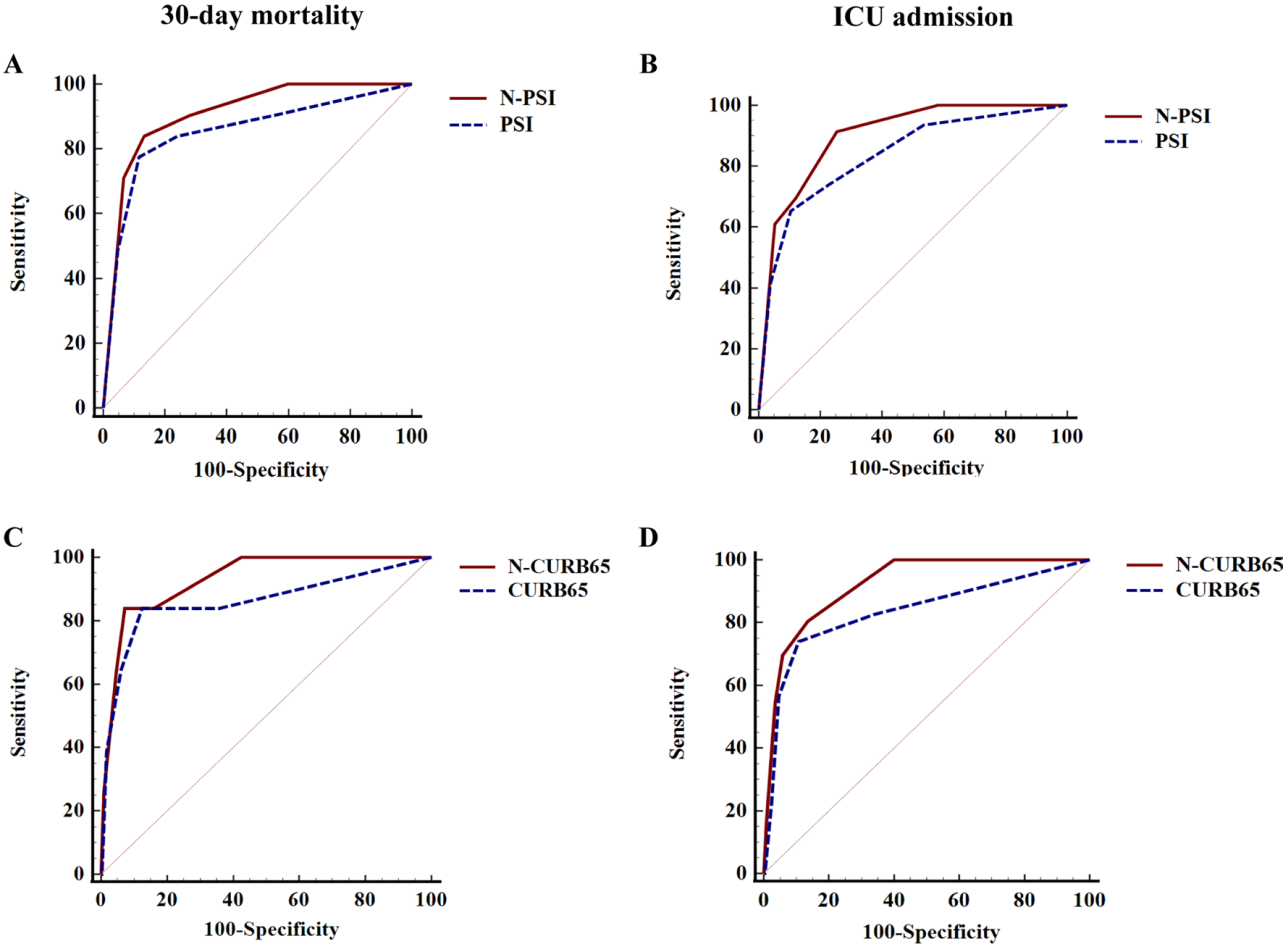
Optimized effect of NMI on the PSI and CURB65 for predicting clinical outcomes. Comparison of ROC curves between N-PSI and PSI for predicting 30-day mortality **A** and ICU admission (**B**). ROC curves between N-CURB65 score and CURB65 score for 30-day mortality **C** and ICU admission (**D**)

**Fig. 4:**
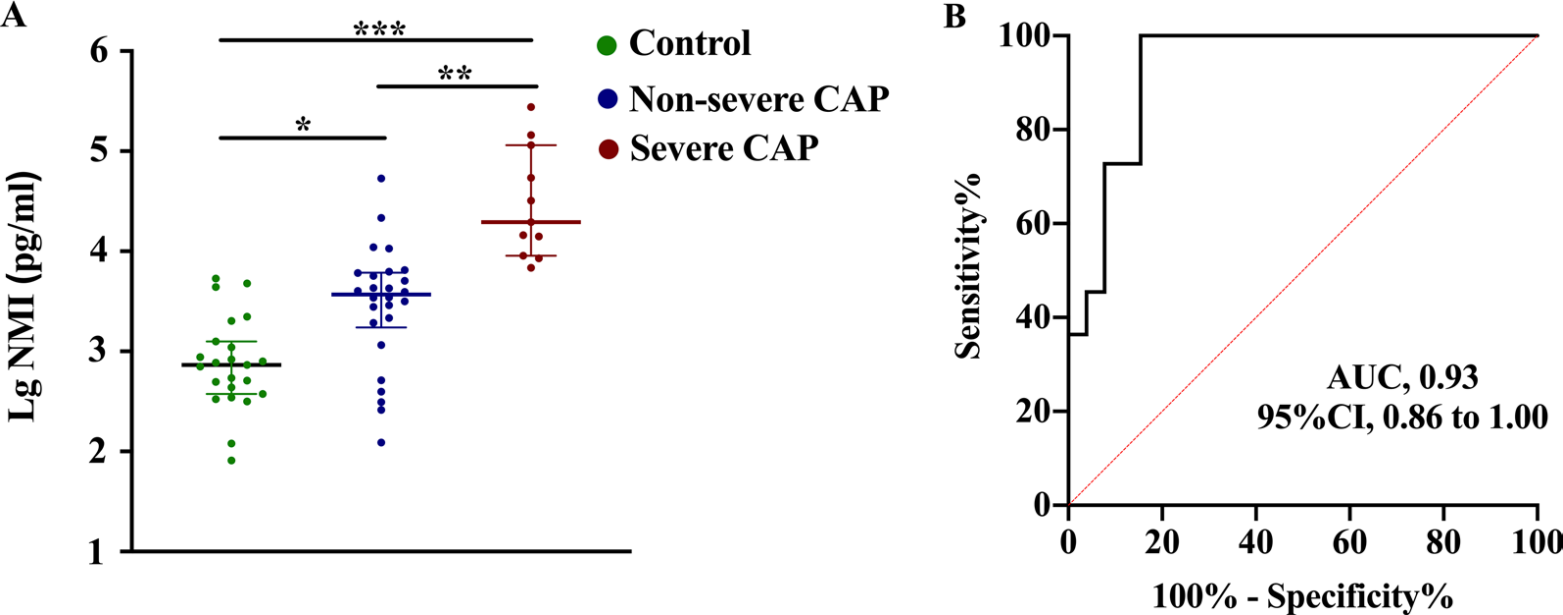
NMI levels in BALF **A** and ROC curve of NMI to predict severe CAP (**B**). Lower and upper lines indicate the 25th and 75th percentiles; middle lines indicate the 50th percentiles. *: *P* < 0.05, **: *P* < 0.01, ***: *P* < 0.001

In addition, we analyzed differences in the incidence of clinical outcomes by NMI cut-off value (55.48 pg/ml) and across different NMI concentration stratification by Kaplan–Meier survival curves. The risk of 30-day mortality and ICU admission increased as the level of NMI rose (*P* < 0.001, Fig. 1A–D). The 30-day mortality rate (37.5%) and ICU admission rate (62.5%) were the highest in patients with NMI > 100 pg/ml, while the 30-day mortality rate and ICU admission rate were 0.4% and 0.8% in patients with NMI < 25 pg/ml, respectively (Fig. 1C, D).

### Serum NMI levels of CAP patients with different risk stratifications

To compare the differences in serum NMI levels of CAP patients with different risk stratifications, we tested serum NMI levels in healthy controls and CAP patients. Levels of NMI in the serum of patients with severe CAP were significantly increased as compared to healthy controls or those with non-severe disease ( *P* < 0.001) (Additional file 1: Fig. S5). We then tested the NMI levels of patients with different classes of the PSI and CURB65 scores. Results showed that NMI levels increased from the low-risk group to high-risk group in both PSI and CURB65 score system (*P* < 0.01), except that there was no difference between the PSI of IV and V (Fig. 2). We also evaluated whether addition of NMI to either the PSI score or the CURB65 score improved their predictive ability. In our cohort, the AUC of the new score system (the N-PSI and N-CURB65 score) for predicting mortality [0.91 (95% CI: 0.86–0.96) and 0.93 (95% CI: 0.89–0.97), respectively] was significantly increased over the scores that did not include the NMI level, and the AUC of new score system for ICU admission [0.90 (95% CI: 0.86–0.94) and 0.92 (95% CI: 0.89–0.96), respectively] was also higher than the scores without NMI (Fig. 3, Table 3).

**Fig. 5:**
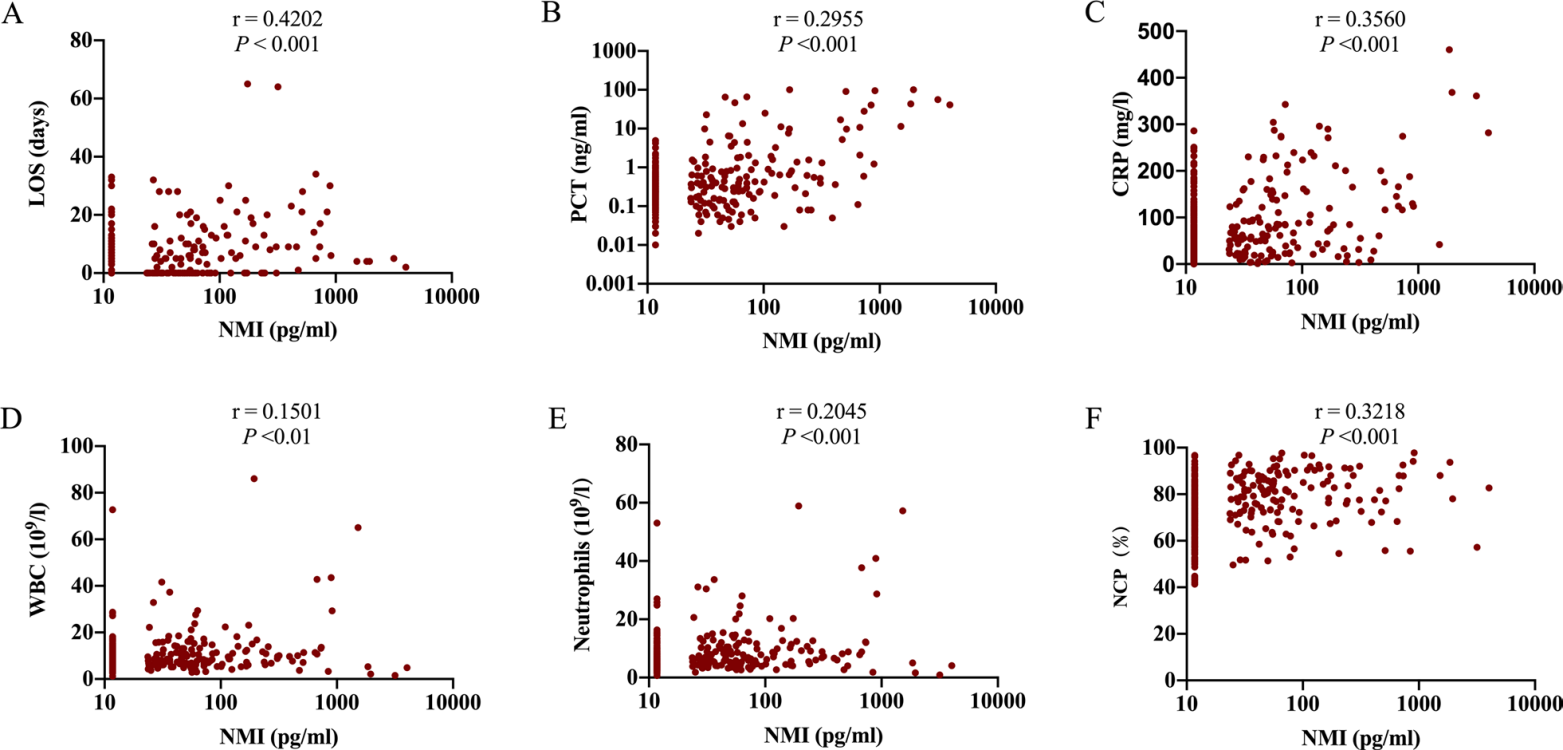
Correlation of NMI levels with LOS and other parameters. Correlation of NMI levels with LOS (**A**), PCT (**B**), CRP (**C**), WBC (**D**), Neutrophils (**E**), and NCP **F** in CAP patients

### NMI levels in BALF

To further evaluate the broad applicability of NMI prediction on CAP severity, we also measured the levels of NMI in BALF of CAP patients and analyzed the difference of NMI concentrations among severe group, non-severe group and non-infected controls. A total of 37 CAP patients were tested, eleven of whom were severe CAP. The concentration of NMI in severe CAP patients [19573 (9032-115254) pg/ml] was significantly higher than that in non-severe CAP group [3690 (1731–6129) pg/ml, *P* < 0.01] and non-infected controls [733.1 (376.7–1254) pg/m, *P* < 0.001], and the AUC for predicting severe CAP was 0.93 (95% CI: 0.86–1.00) (Fig. 4).

### Correlation of NMI with LOS and other indicators

To validate the relationship between serum NMI levels and current severity assessment indicators of CAP, we evaluated the correlation of NMI with LOS, PCT, CRP, WBC, neutrophil count and NCP using Spearman correlation coefficient. As shown in Fig. 5, NMI was significantly correlated with LOS (correlation coefficient r = 0.4202, *P* < 0.001), and also with PCT, CRP, WBC, neutrophils and NCP.

## Discussion

The early stratification of CAP patients helps clinicians formulate a diagnosis and treatment plan and optimize hospital resource use [28]. NMI is a regulator involved in macrophage-induced inflammation. In this study, we tested NMI levels in patients with CAP to explore whether there might be a role for measuring NMI in predicting the severity of CAP. We measured NMI levels in both serum and BALF of CAP patients and analyzed its correlation with 30-day mortality and ICU admission. We compared the efficiency of NMI levels in predicting CAP severity with other classical CAP severity score systems and biomarkers. Our results showed that NMI is a novel biomarker reflecting the severity of CAP patients.

Many parameters have been studied and widely applied to assess the severity of CAP patients, among which the PSI and CURB65 score are the most widely used [9]. The PSI score can accurately predict the 30-day mortality rate; however, the complexity of its 20 variables limits its clinical application. As simple as CURB-65 is, it underestimates the potential severity of pneumonia in young patients and can mistake elderly CAP patients as severe CAP patients [9]. An expanded version CURB-65 improved the recognition of patients with severe CAP compared with CURB-65, but there remains room for improvement [29]. The addition of specific biomarkers may further improve the predictive ability of these scores in clinical outcomes [30]. A more convenient, highly efficient, and earlier recognition score system is urgently needed.

Some biomarkers were showed to be related to CAP, such as PCT, CRP, proADM, and c-terminal vasopressin (copeptin) [14, 15, 31, 32], and they play an important role in estimating the severity, treatment, discontinuation, and etiology in the management of CAP patients [33]. PCT can help determine whether antibiotics should be used or stopped in lower respiratory tract infections [33]. However, measuring PCT does not improve the predictive ability of the PSI/CURB65 score [34] and is insufficient to distinguish bacterial from viral infection [35]. CRP shows only moderate predictive values for mortality of CAP [15, 34]. Although CRP indicates bacterial pneumonia, their value is limited in predicting the severity of CAP in the early stage [36, 37]. Some new biomarkers like proADM and copeptin may be useful to infer mortality and severity of CAP, but further verification is needed [9, 38].

In our study, measurement of NMI concentration at admission was superior in assessing the severity and risk of death of CAP as compared to the serum biomarkers above. We found a higher comprehensive efficiency of NMI than CRP and PCT in predicting 30-day mortality and ICU admission in CAP patients. Serum NMI level of patients with severe CAP was also significantly higher than that of non-severe CAP patients on the day of admission, suggesting that NMI may be an early indicator of the severity of CAP. Finally, the AUC of the PSI and CURB65 score was significantly increased by the addition of NMI for both for 30-day mortality and ICU admission. These results indicate the potential value of NMI in the clinical assessment of CAP severity.

Previous studies have already shown that NMI is associated with various inflammatory diseases while the serum NMI level was low in healthy people or mice without infection [18, 23, 24]. Xiahou et al. found that serum NMI levels significantly increased in sepsis patients and were associated with mortality [24]. Wu et al. showed that NMI expression in human lung A549 cells was increased after H3N2 SIV infection. NMI levels also were elevated in serum and liver tissue of patients with hepatitis B virus-related acute-to-chronic liver failure and the concentrations of NMI decreased when in convalescent stage of disease [18]. Our study showed that the NMI levels in both serum and BALF significantly increased in CAP and that NMI levels were positively correlated with mortality and ICU admission. Together, these results are consistent with the idea that NMI expression in both serum and local tissue are increased in infectious diseases, and might be related to the prognosis of the disease. Interference with NMI expression may relieves inflammation and improve prognosis. One set of experiments confirmed that NMI and interferon-induced protein 35 knockout mice had reduced inflammation and mortality in a sepsis model [24]. Similarly, apoptosis induced by foot and mouth disease virus was significantly inhibited after silencing NMI expression [39]. Therefore, the NMI might be used not only as a predictor of the severity, but also as a therapeutic target of CAP patients in the future.

## Conclusion

In this cohort study, we tested the NMI levels in serum and BALF of CAP patients and found that NMI levels are increased in severe CAP patients. We demonstrated that NMI can act as a novel predictive biomarker for the 30-day mortality and ICU admission, and is a useful measure for early risk stratification and accurate clinical decision-making in CAP.

## Supplementary Information

**Additional file 1.** Additional tables and figures.

## Data Availability

The datasets used and/or analyzed during the current study are available from the corresponding author on reasonable request.

## Abbreviations

AUC: Area under the curve
BALF: Bronchoalveolar lavage fluid
CAP: Community-acquired pneumonia
CRP: C-reactive protein
ICU: Intense care unit
LOS: Length of stay
NCP: Neutrophil count percentage
NMI: N-myc and STAT interactor
PCT: Procalcitonin
proADM: Proadrenomedullin
PSI: Pneumonia severity index
ROC: Receiver operating characteristic
WBC: White blood cell
