# Supplementary material for: N-myc and STAT interactor is a novel biomarker of severity in community-acquired pneumonia: a prospective study
Source: Respir Res. 2022 Sep 19;23:253. doi: 10.1186/s12931-022-02139-x (PMC9483521; doi:10.1186/s12931-022-02139-x)
Supplement: Supplementary file 1 — Additional file 1. Additional tables and figures. [file 12931_2022_2139_MOESM1_ESM.docx]

**Additional file 1: Table S1.** Baseline characteristics and outcomes of CAP patients from whom the BALF was collected.

| **Characteristics** | **Patients with CAP (n=37)** | |
| --- | --- | --- |
| **Demographic characteristics** | | |
| Age (years) | | 54 (35–66) |
| Males | | 24 (64.9) |
| **Coexisting illnesses** | | |
| Liver disease | | 8 (21.6) |
| Diabetes mellitus | | 7 (18.9) |
| Hypertensive heart disease | | 4 (10.8) |
| Cerebrovascular disease | | 4 (10.8) |
| Renal dysfunction | | 3 (8.1) |
| Chronic obstructive pulmonary disease | | 2 (5.4) |
| Congestive heart failure | | 1 (2.7) |
| Neoplastic disease | | 1 (2.7) |
| **Antibiotic pretreatment** | | 17 (45.9) |
| **Laboratory findings** | | |
| PCT (ng/ml) | | 0.37 (0.05-0.80) |
| CRP (mg/l) | | 23.20 (6.35-97.50) |
| WBC (10^9/l) | | 7.35 (6.03-10.83) |
| Neutrophils (10^9/l) | | 5.88 (3.78-9.30) |
| NCP (%) | | 75.50 (60.43-86.10) |
| **Radiographic findings** | | |
| Pleural effusion | | 17 (45.9) |
| Multilobar infection | | 22 (59.5) |
| **PSI class** | | |
| I-III | | 30 (81.1) |
| IV | | 2 (5.4) |
| V | | 5 (13.5) |
| **CURB65 score class** | | |
| 0–1 | | 27 (73.0) |
| 2 | | 4 (10.8) |
| 3-5 | | 6 (16.2) |
| **Clinical outcomes** | | |
| 30-day mortality | | 8 (21.6) |
| ICU admission | | 10 (27.0) |

Data are presented as median (interquartile range) or n (%). Bronchoalveolar lavage (BAL) was performed in CAP patients . We only collected the first BALF as a sample if patients who have undergone bronchoscopy multiple times.

**Additional file 1: Table S2.** Baseline characteristics of controls from whom the BALF was collected.

| **Characteristics** | **Controls (n = 23)** | |
| --- | --- | --- |
| **Demographic characteristics** | | |
| Age (years) | | 59 (53–68) |
| Males | | 11 (47.8) |
| **Reasons for Bronchoscopy** | | |
| Lung tumor | | 11 (47.8) |
| Lung nodules | | 5 (21.7) |
| Pulmonary sarcoidosis | | 4 (17.4) |
| Interstitial lung disease | | 2 (8.7) |
| Chronic cough | | 1 (4.3) |
| **Laboratory findings** | | |
| CRP (mg/l) | | 4.75 (1.75-8.35) |
| WBC (10^9/l) | | 6.10 (4.40-8.60) |
| Neutrophils (10^9/l) | | 4.23 (2.70-6.86) |
| NCP (%) | | 66.60 (62.20-79.80) |

Data are presented as median (interquartile range) or n (%).

**Additional file 1: Table S3.** Comparison of baseline characteristics and outcomes between survivors and non-survivors of CAP patients from whom the serum was collected.

| **Characteristics** | **Survivors (n=363)** | **Non-survivors (n=31)** |
| --- | --- | --- |
| **Demographic characteristics** | |  |
| Age (years) | 52 (30–66) | 66 (56-78) |
| Males | 213 (58.7) | 22 (71.0) |
| **Coexisting illnesses** | |  |
| Hypertensive heart disease | 52 (14.3) | 16 (51.6) |
| Diabetes mellitus | 29 (8.0) | 7 (22.6) |
| Chronic obstructive pulmonary disease | 13 (3.6) | 4 (12.9) |
| Liver disease | 10 (2.8) | 4 (12.9) |
| Coronary artery disease | 9 (2.5) | 2 (6.5) |
| Renal dysfunction | 4 (1.1) | 5 (16.1) |
| Congestive heart failure | 5 (1.4) | 3 (9.7) |
| Cerebrovascular disease | 5 (1.4) | 3 (9.7) |
| Neoplastic disease | 6 (1.7) | 2 (6.5) |
| **Antibiotic pretreatment** | 86 (23.7) | 21 (67.7) |
| **Laboratory findings** | |  |
| PCT (ng/ml) | 0.25 (0.14-0.51) | 1.59 (0.30-13.34) |
| CRP (mg/l) | 42.10 (14.10-92.60) | 155.50 (61.20-274.40) |
| WBC (10^9/l) | 7.90 (6.10-11.10) | 9.50 (6.60-15.00) |
| Neutrophils (10^9/l) | 6.00 (4.10-8.80) | 8.30 (5.80-12.50) |
| NCP (%) | 74.00 (66.40-82.30) | 86.30 (78.30-90.40) |
| **Radiographic findings** | |  |
| Pleural effusion | 83 (22.9) | 24 (77.4) |
| Multilobar infection | 116 (32.0) | 30 (96.8) |
| **PSI class** | |  |
| I-III | 321 (88.4) | 7 (22.6) |
| IV | 25 (6.9) | 9 (29.0) |
| V | 17 (4.7) | 15 (48.4) |
| **CURB65 score class** | |  |
| 0–1 | 318 (87.6) | 5 (16.1) |
| 2 | 23 (6.3) | 6 (19.4) |
| 3-5 | 22 (6.1) | 20 (64.5) |
| **Clinical outcomes** | |  |
| ICU admission | 20 (5.5) | 26 (83.9) |

Data are presented as median (interquartile range) or n (%).

**Additional file 1: Table S4.** Comparison of baseline characteristics and outcomes between ICU-admitted and non-ICU-admitted CAP patients from whom the serum was collected.

| **Characteristics** | **Non-ICU-admitted patients**  **(n=348)** | **ICU-admitted patients**  **(n=46)** |
| --- | --- | --- |
| **Demographic characteristics** | |  |
| Age (years) | 51 (30–65) | 66.5 (55–74.25) |
| Males | 201 (57.8) | 34 (73.9) |
| **Coexisting illnesses** | |  |
| Hypertensive heart disease | 50 (14.4) | 18 (39.1) |
| Diabetes mellitus | 25 (7.2) | 11 (23.9) |
| Chronic obstructive pulmonary disease  disease | 10 (2.9) | 7 (15.2) |
| Liver disease | 7 (2.0) | 7 (15.2) |
| Coronary artery disease | 9 (2.6) | 2 (4.3) |
| Renal dysfunction | 3 (0.9) | 6 (13.0) |
| Congestive heart failure | 4 (1.1) | 4 (8.7) |
| Cerebrovascular disease | 3 (0.9) | 5 (10.9) |
| Neoplastic disease | 5 (1.4) | 3 (6.5) |
| **Antibiotic pretreatment** | 72 (20.7) | 35 (76.1) |
| **Laboratory findings** | |  |
| PCT (ng/ml) | 0.25 (0.13-0.48) | 1.72 (0.40-25.76) |
| CRP (mg/l) | 39.25 (12.83-90.2) | 126.95 (59.4-214.05) |
| WBC (10^9/l) | 7.90 (6.10-10.90) | 9.15 (6.33-17.88) |
| Neutrophils (10^9/l) | 5.95 (4.10-8.58) | 7.85 (4.68-15.08) |
| NCP (%) | 73.90 (66.40-81.98) | 86.40 (77.58-90.55) |
| **Radiographic findings** | |  |
| Pleural effusion | 70 (20.1) | 37 (80.4) |
| Multilobar infection | 105 (30.2) | 41 (89.1) |
| **PSI class** | |  |
| I-III | 312 (89.7) | 16 (34.8) |
| IV | 23 (6.6) | 11 (23.9) |
| V | 13 (3.7) | 19 (41.3) |
| **CURB65 score class** | |  |
| 0–1 | 311 (89.4) | 12 (26.1) |
| 2 | 21 (6.0) | 8 (17.4) |
| 3-5 | 16 (4.6) | 26 (56.5) |
| **Clinical outcomes** | |  |
| 30-day mortality | 5 (1.4) | 26 (56.5) |

Data are presented as median (interquartile range) or n (%).

**Additional file 1: Figure S1.** Flow diagram for serum collection from CAP patients and healthy volunteers

**
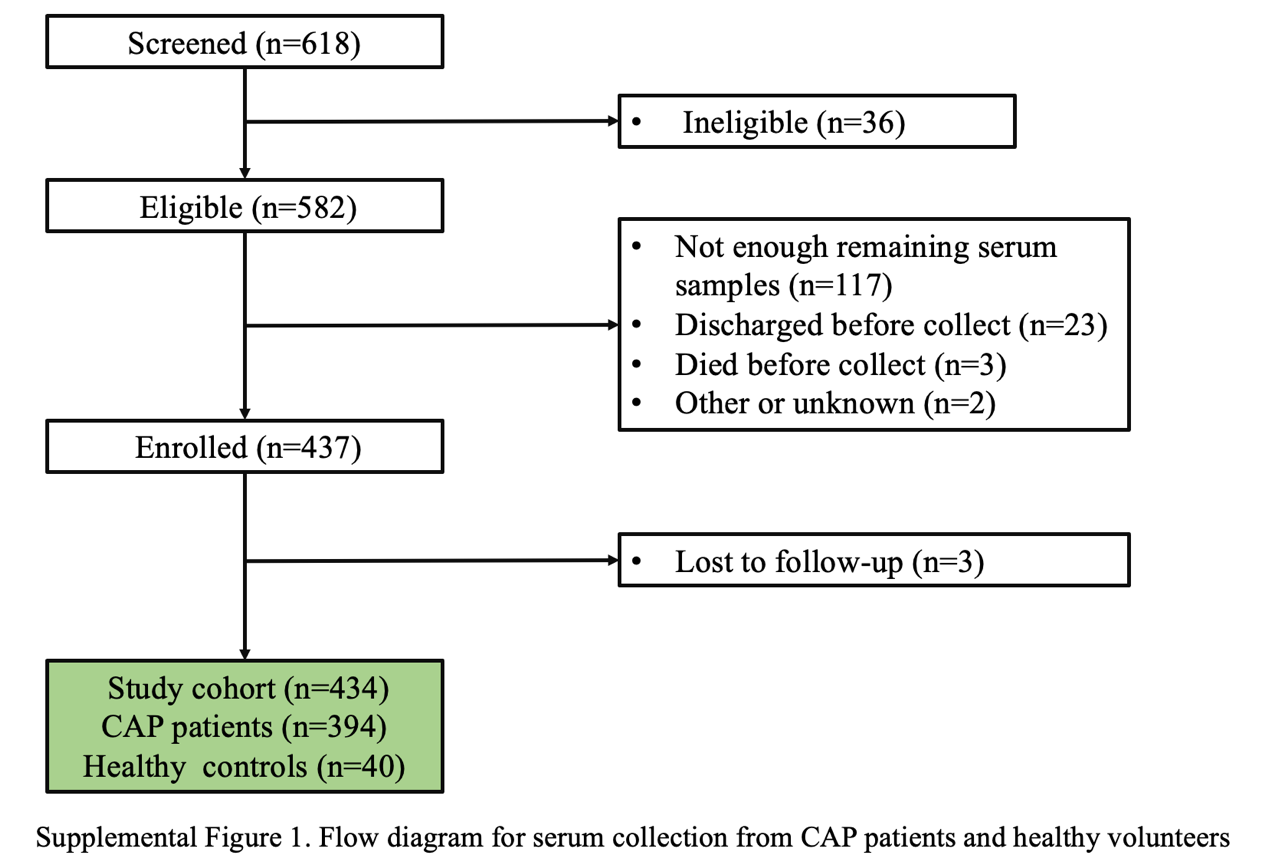
**

**Additional file 1: Figure S2.** Flow diagram for BALF collection from CAP patients and controls

**
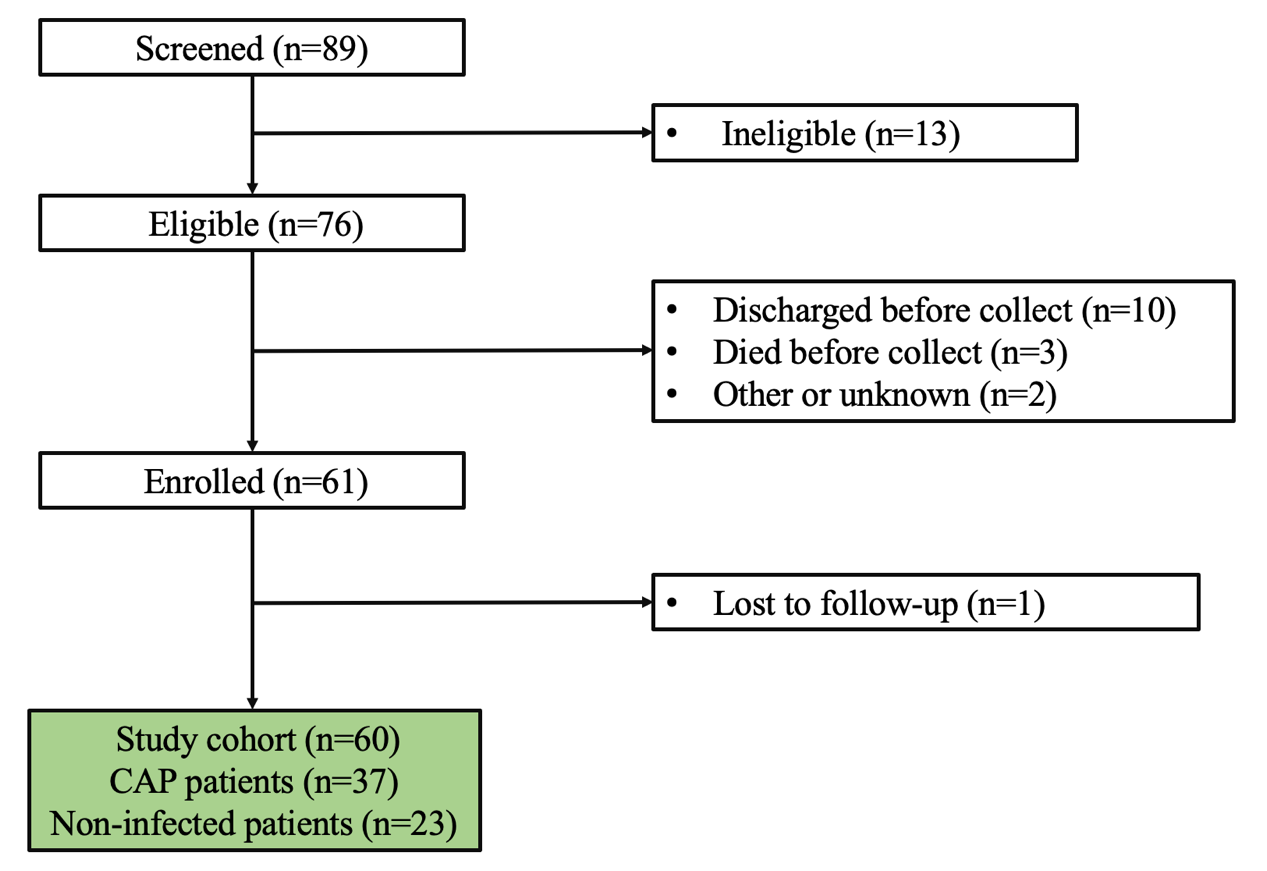
**

**Additional file 1: Figure S3.** Differences in levels and ROC curve for predicting mortality, using NMI (A-B), PCT (C-D), CRP (E-F), WBC (G-H), Neutrophils (I-J) and NCP (K-L) between survivors and non-survivors in adult CAP patients. Lower and upper lines indicate the 25th and 75th percentiles; middle lines indicate the 50th percentiles. **: *P* < 0.01, ***: *P* < 0.001.


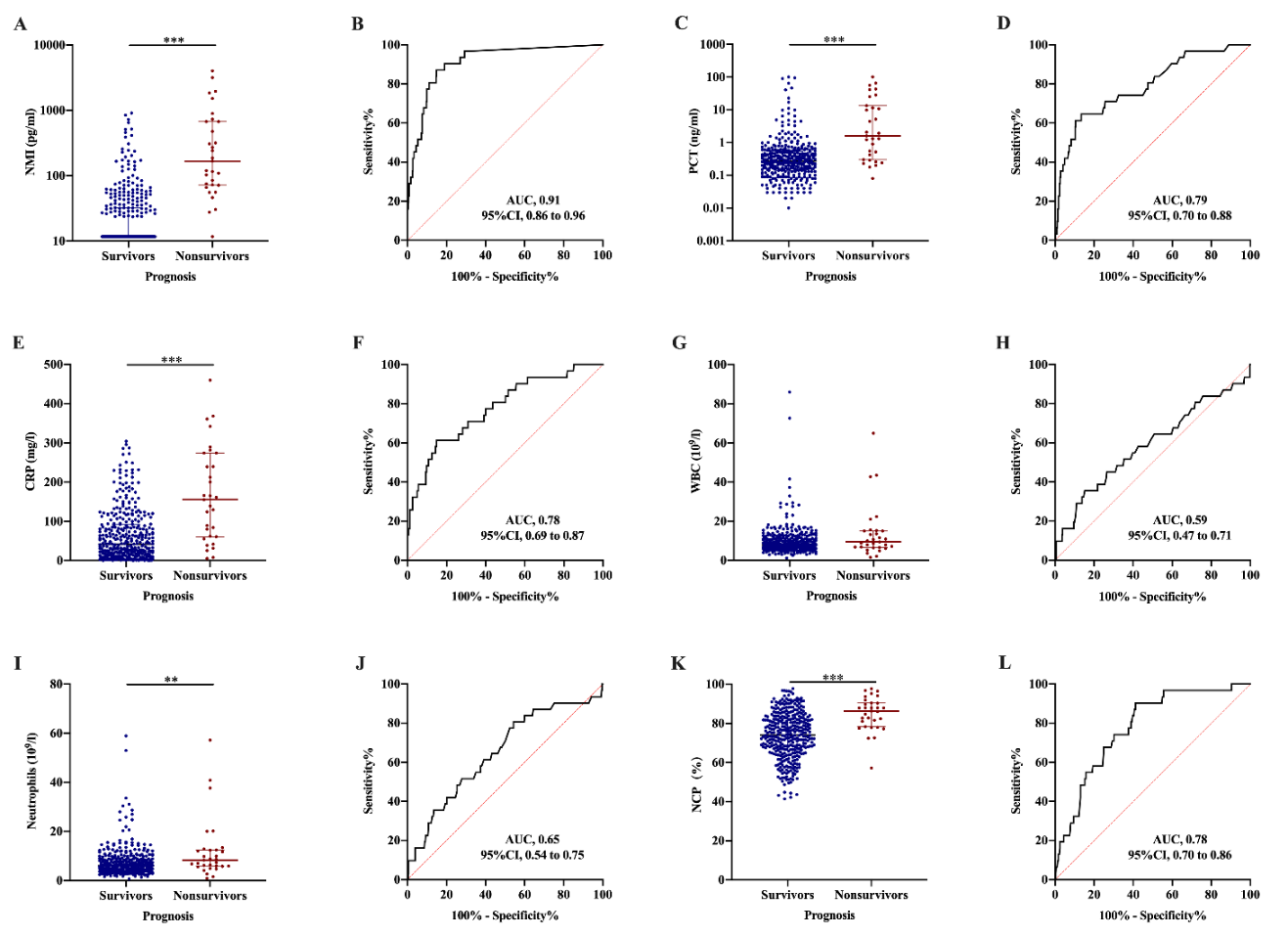


**Additional file 1:** Differences in levels and ROC curve of NMI (A-B), PCT (C-D), CRP (E-F), WBC (G-H), Neutrophils (I-J) and NCP (K-L) between non-ICU and ICU CAP patients. Lower and upper lines indicate the 25th and 75th percentiles; middle lines indicate the 50th percentiles. **: *P* < 0.01, ***: *P* < 0.001.


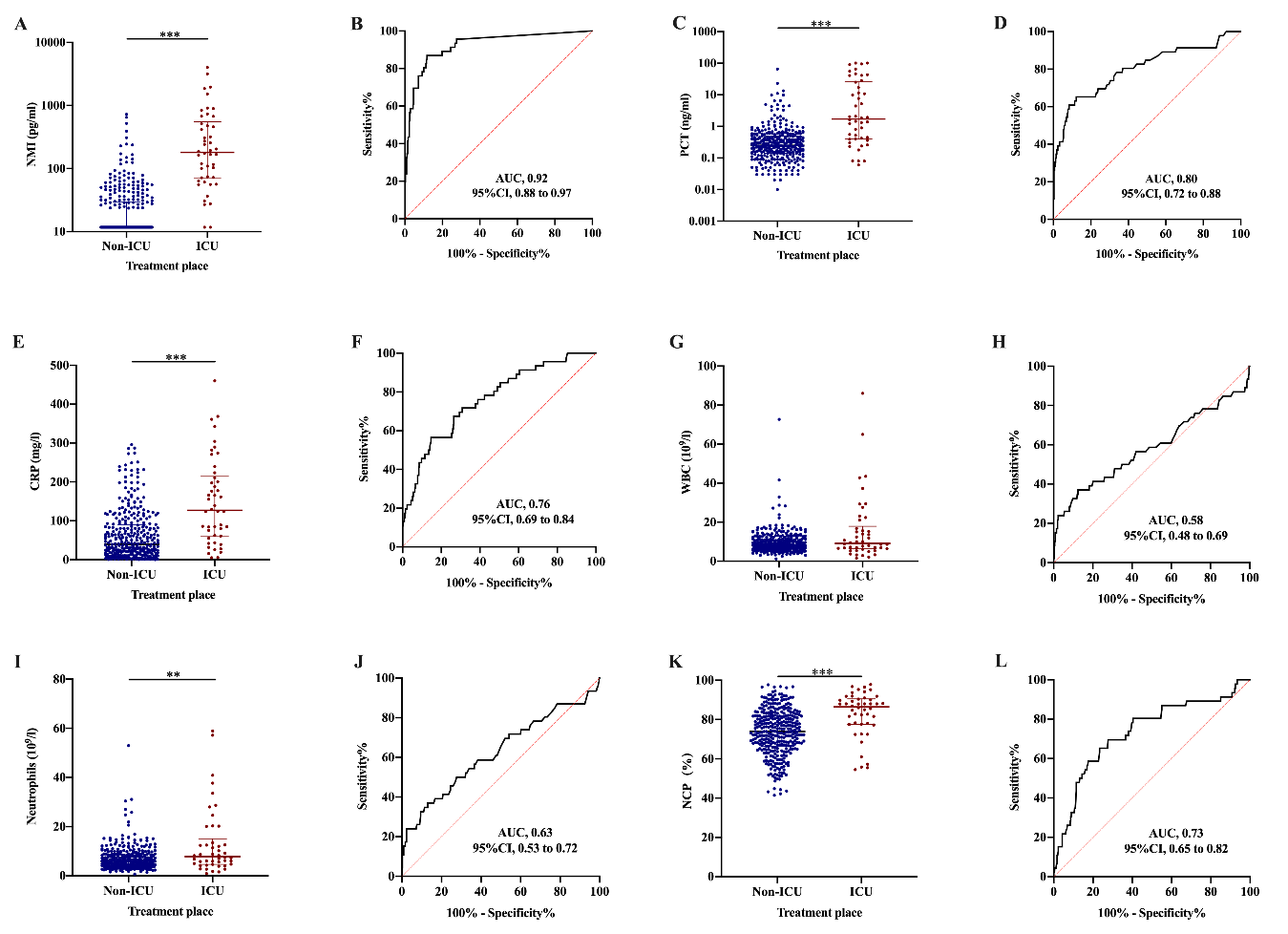


**Additional file 1: Figure S5.** Difference of NMI levels in serum among healthy controls, non-severe and severe CAP patients. In 62.18% (245/394) of CAP patients and 65% (26/40) of healthy controls, serum NMI levels determined by ELISA were below the minimum detection threshold of the standard curve (23.44pg/ml) given by the instructions. The samples below the lower limit were uniformly quantified as half of the minimum detection threshold (11.72pg/ml).
